# Relaxation therapy as a treatment for tics in patients with Tourette syndrome: a systematic literature review

**DOI:** 10.1007/s10072-019-04207-5

**Published:** 2019-12-23

**Authors:** Florence Tilling, Andrea E. Cavanna

**Affiliations:** 1 Department of Neuropsychiatry, BSMHFT and University of Birmingham, Birmingham, UK; 2 School of Life and Health Sciences, Aston University, Birmingham, UK; 3 University College London and Institute of Neurology, London, UK

**Keywords:** Behavioural therapy, Relaxation, Tics, Tourette syndrome

## Abstract

**Background:**

Tourette syndrome (TS) is a neurodevelopmental condition characterized by the presence of multiple motor and phonic tics, often associated with co-morbid behavioural problems. Tics can be modulated by environmental factors and are characteristically exacerbated by psychological stress, among other factors. This observation has led to the development of specific behavioural treatment strategies, including relaxation therapy.

**Objective:**

This review aimed to assess the efficacy of relaxation therapy to control or reduce tic symptoms in patients with TS.

**Methods:**

We conducted a systematic literature review of original studies on the major scientific databases, including Medline, EMBASE, and PsycInfo, according to the standards outlined in the Preferred Reporting Items for Systematic Reviews and Meta-Analyses (PRISMA) guidelines. Outcomes measures included both tic severity and tic frequency.

**Results:**

Our literature search identified three controlled trials, with a total number of 40 participants (range: 6–18 participants). In all three studies, relaxation therapy decreased the severity and/or the frequency of tic symptoms. However, the only trial comparing relaxation therapy to two other behavioural techniques found relaxation therapy to be the least effective intervention, as it reduced the number of tics by 32% compared to 44% with self-monitoring and 55% with habit reversal.

**Discussion:**

The results of this systematic literature review provide initial evidence for the use of relaxation therapy as a behavioural treatment intervention for tics in patients with TS. Caution is needed in the interpretation of these findings, because the reviewed trials had small sample sizes and there was high heterogeneity across the study protocols.

## Introduction

Tourette syndrome (TS) is a neurodevelopmental disorder characterized by the chronic presence of motor and phonic tics. Tics are defined as sudden, involuntary, repetitive, non-rhythmic movements (motor tics) or vocalizations (phonic tics) [1, 2]. Tics are preceded by sensory experiences referred to as ‘premonitory urges’ and are modulated by environmental factors [2, 3]. Based on clinical observations, stress has long been associated with tic exacerbations, contributing to the characteristic ‘waxing and waning’ course of TS [4]. Specifically, the results of multiple studies have suggested the existence of a relationship between tic severity and both psychological stress [5, 6] and physical stress [7, 8]. Moreover, self-report measures of patients with TS have pointed towards a correlation between tic frequency and daily life stress [9]. From the neurobiological point of view, there is evidence of overactivation of the hypothalamic-pituitary-adrenal axis in patients with TS [10]. In consideration of these findings, a number of behavioural treatment interventions targeting patients’ stress have been implemented in order to decrease tic severity and frequency [11]. We set out to conduct a systematic literature review on the effectiveness of relaxation therapy in controlling or reducing tics in patients with TS compared to other treatment types or no treatment. We aimed to fill an important gap in our knowledge about TS, as information about the effectiveness of relaxation therapy can inform clinicians about the choice of appropriate treatment interventions for patients with tics.

## Methods

This systematic literature review was conducted in accordance with the Preferred Reporting Items for Systematic Reviews and Meta-Analyses (PRISMA) guidelines [12], used in conjunction with the Explanation and Elaboration document [13]. We included in our review all studies using a form of relaxation therapy as the main treatment intervention for tics in patients with a diagnosis of TS or other persistent tic disorders. The relaxation therapies included were those in which individuals were taught a specific relaxation technique by a clinician or expert: relaxation training, hypnosis and/or relaxation with mental imagery, yoga, and mindfulness based stress reduction [14]. Studies using behavioural interventions that were not predominantly based on muscular relaxation were excluded, as well as studies that did not specify the details of the relaxation technique. Studies using Habit Reversal Training were also excluded, as relaxation training is not the core element of this behavioural technique for tic control. We included both studies using another form of behavioural therapy as comparator and studies using no comparator. The outcomes of interest were the severity and frequency of tics, assessed with validated measures.

The searches were conducted on five databases: MEDLINE, Embase, PsycInfo, and NCBI PubMed. The search terms were as follows: ‘Tourette*’ OR ‘Tic*’ AND ‘Relaxation’ OR ‘Stress reduction’ OR ‘Stress relief’. For comprehensiveness, the reference lists of eligible articles were also screened to identify any relevant articles. Google scholar was used to search for the grey literature. Since a few of the reviewed studies were published in the same scientific journals, the contents of the ‘Journal of Behavior Therapy and Experimental Psychiatry’ and ‘Behaviour Research and Therapy’ were manually searched for any further relevant articles. We limited our search to articles published in English language, but there were no chronological, geographic, or demographic limitations to the inclusion of studies. Both qualitative and quantitative study designs were included for review. We excluded studies in which medications were used, including anti-anxiety agents, muscle relaxants, and alpha-2 adrenergic agonists, which modulate noradrenaline signalling, a neurotransmitter normally produced in response to stress [15–17]. The Crowe Critical Appraisal Tool (CCAT) was used to assess the quality of the selected studies, and any studies scoring less than 30% were excluded from the review [18, 19].

## Results

**Fig. 1:**
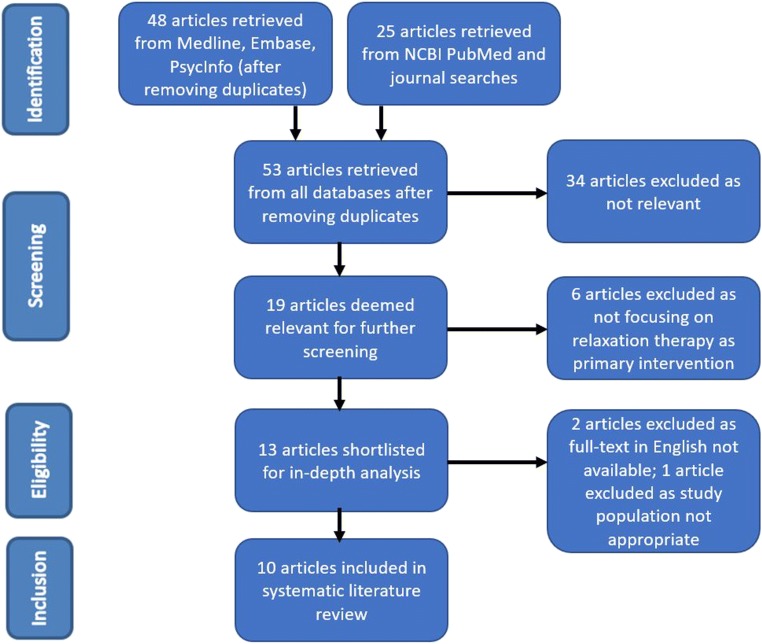
PRISMA flow diagram outlining the identification, screening, assessment for eligibility, and inclusion of studies in the present review

Our systematic literature search yielded a total of 53 articles, after removal of duplicates. Of these, 13 were considered relevant to the review and their full texts were inspected. A further two studies were excluded because the English full text was not available; one study was excluded because its participants did not have a formal diagnosis of TS or other persistent tic disorders [20]. One study retrieved by manually searching the contents of relevant journals was excluded as it focused on a different behavioural technique for tic control (massed practice) [21]. A total of 10 results met all the inclusion criteria. This methodology is displayed in the flow diagram (Fig. 1).

Three original studies on relaxation therapy as a treatment for tics were included in the present review (Table 1): a randomized controlled trial involving 16 participants with a diagnosis of TS in which relaxation training was compared to a control arm of relaxation involving music and sitting quietly [23]; a single arm study involving 18 participants on mindfulness-based stress reduction consisting of sitting meditation, walking meditation, yoga, and body scan [22]; a controlled trial in which 6 participants received three different behavioural interventions [24]. As the studies had different comparators and outcome measures, a meta-analysis could not be performed [25].

**Table 1:** Summary of studies on relaxation therapy as a treatment for tics (ranked by study quality, as assessed by CCAT score)

Article	Intervention(s)	Participants	Study type	Procedure	Findings	Comments	CCAT score (%)
Reese et al. 2014 [22]	Mindfulness-based stress reduction	*N* = 18 16–67 years old (mean 34.8) Diagnosis of TS (*N* = 17) and persistent motor tic disorder (*N* = 1)	Open trial	Participants had 8 weekly 2-h classes and one 4-h retreat. Mindfulness-based stress reduction consisted of sitting meditation, body scan, yoga, and walking meditation.	Tic severity significantly decreased with treatment intervention, including at 1-month follow-up.	No control comparator. Possible selection bias	80
Bergin et al. 1998 [23]	Relaxation training	*N* = 16 (7: intervention vs 9: minimal treatment) 7–18 years old (mean 11.3) 19 males and 4 females (5 males and 2 females dropped out before completion) Diagnosis of TS	Randomized controlled trial	Participants had 6 weekly 1-h training sessions with follow-up at 6 weeks and 3 months. Intervention group received direct training on awareness, diaphragmatic breathing, behavioural and applied relaxation techniques, and biofeedback. Control group listened to cassettes of music or sounds and were asked to sit quietly. Both groups were given positive reinforcement	No significant difference between the groups. Improvement in tic severity was noted with both interventions at 6-week and 3-month follow-up. No improvement in behavioural measures in either group	Control group also received relaxation therapy, although not formally taught	70
Peterson and Azrin 1992 [24]	Relaxation training, self-monitoring, and habit reversal	*N* = 6 4 adults, 2 children All males Diagnosis of TS	Controlled trial	Participants were taught 3 behavioural techniques to control their tics (relaxation training, self-monitoring, habit reversal) and were filmed both at baseline and when practicing each of their newly learned techniques	Relaxation training reduced the total number of tics by 32%, self-monitoring by 44%, habit reversal by 55%. No statistical difference between the three interventions. Relaxation training resulted in the largest effect of 60% tic reduction in 1 participant	Small sample size. Short treatment trials (10 min). No assessment of long-term efficacy of interventions	63

*CCAT* Crowe Critical Appraisal Tool

A further seven articles presenting individual case studies and case series were included in the review for separate qualitative analysis (Table 2).

**Table 2:** Summary of individual case studies and case series on relaxation therapy as a treatment for tics (ranked by study quality, as assessed by CCAT score)

Article	Intervention(s)	Participant(s)	Study type	Procedure	Findings	Comments	CCAT score (%)
Michultka and Blanchard 1989 [26]	Relaxation training and desensitization	*N* = 1 19 years old Male Diagnosis of TS	Case study	Participant had training in progressive muscle relaxation and desensitization by imagining stressful situations and engaging muscle relaxation. Pre-treatment and post-treatment assessment	Whilst engaged in relaxation technique, no tics were present. Distress, frequency, and intensity decreased by over 40% according to both subject and parental monitoring	Idiosyncratic response with mild symptoms does not allow for generalization	70
Zarkowska et al. 1989 [27]	Relaxation training	*N* = 1 13 years old Female Diagnosis of TS and mental retardation	Case study	Participant had 3 daily 10-min sessions of relaxation therapy for 5 weeks, subsequently reduced to 2 daily sessions for 3 weeks. Participant was also instructed to relax throughout the day	During relaxation training, tic frequency decreased, but returned to baseline immediately afterwards	Idiosyncratic response with lack of active involvement does not allow for generalization	68
James-Roberts and Powell 1978 [28]	Relaxation therapy versus Massed practice	*N* = 1 45 years old Male Diagnosis of tic disorder	Case study	Participant had 12 sessions of relaxation therapy (self-hypnosis) and 12 sessions of massed practice (voluntary repetition of an existing tic). Assessment of tic severity at the beginning and at the end of each session	Massed practice was found to be significantly more effective than relaxation therapy. Whilst engaged in relaxation technique no tics were present, but tics increased after the session	Idiosyncratic response does not allow for generalization	63
Turpin and Powell 1983 [29]	Relaxation training versus massed practice	*N* = 3 27 years old (*N* = 2 females), 36 years old (*N* = 1 male) Diagnosis of TS (*N* = 2) and persistent motor tic disorder (*N* = 1)	Case series	Relaxation therapy consisted of an 18-min tape instructing muscle relaxation. Massed practice consisted of voluntary repetition of an existing tic for 5 min and resting for 1 min, repeated 3 times per session. Both interventions were accompanied by 30 min of home practice daily	One participant reported a decrease in tic frequency after both interventions (higher with relaxation therapy than massed practice). One participant experienced no change at follow-up. One participant experienced an increase in tics after both treatments	No standardized treatment intervention. Different follow-up times	63
Kohen and Botts 1986 [30]	Relaxation/mental imagery	*N* = 4 6–10 years old Diagnosis of TS	Case series	Participants had training in Relaxation/mental imagery technique (hypnotic state of mind and progressive relaxation of the body) and were encouraged to practice at home with use of audiotape. Follow-up for a minimum of 24 months (*N* = 3, good compliance) and for 30 months (*N* = 1, poor compliance)	For all participants, the ability to relax and control their bodies appeared to decrease their tics. For 3 participants, the positive effects were maintained at follow-up	The technique included an element of self-control	48
Culbertson 1989 [31]	Relaxation and hypnotherapy	*N* = 1 16 years old Male Diagnosis of TS	Case study	Participant had 9 sessions of hypnotherapy (progressive muscle relaxation, Spiegel’s eye roll, imagery) over a period of 6 months. Follow-up at 6 months after intervention	Tics were reduced to almost none, with reappearance only when quarrelling	Idiosyncratic response does not allow for generalization	40
Friedman 1980 [32]	Relaxation training and self-control techniques	*N* = 1 11 years old Female Diagnosis of TS	Case study	Participant had 15 sessions (including self-control techniques to prevent coprolalia) over a period of 4 months. Follow-up at 4 and 18 months after intervention	Coprolalia improved significantly. Both participant and parents reported improvement of symptoms at follow-up, with tics only apparent with stress	Idiosyncratic response does not allow for generalization. Participant had pharmacotherapy before follow-up	35

*CCAT* Crowe Critical Appraisal Tool

## Discussion

We conducted a systematic literature review on the effectiveness of relaxation therapy as a treatment for tics in patients with TS, retrieving three original studies that recruited a total of 40 participants. In each of the three controlled trials included in the present review, relaxation therapy decreased the severity and/or the frequency of tics in the study population, therefore supporting its use in patients with TS. However, the only trial comparing relaxation therapy to two other behavioural techniques for tic control found relaxation therapy to be the least effective intervention, as it reduced the number of tics by 32%, compared to 44% with self-monitoring and 55% with habit reversal [24]. Moreover, one of the reviewed trials did not show a statistical difference between the expert-taught relaxation intervention arm and the control group [23]. In this study, both the intervention group and the control group received a form of relaxation training and both groups reported an improvement, although the participants in the control group were not taught a recognized technique for relaxation. The findings of the third study showed a significant improvement in tic severity, however in this trial there was no control group or comparator, resulting in increased risk of bias and decreased reliability [22].

Four of the seven single case studies and case series on the effectiveness of relaxation therapy supported its use for the treatment of tics. Specifically, 7 of the 12 cases (58%) individually described in the literature reported positive results. One of the participants included in a case series dropped out before follow-up assessment; however, she had reported an initial improvement before she dropped out [33]. Overall, the reviewed case studies and case series supported the use of relaxation therapy, although the level of evidence was relatively low, leading to a large margin of error.

Relaxation therapy is currently used as a component of recommended behavioural treatments for tics, such as Habit Reversal Training and the wider Comprehensive Behavioral Intervention for Tics [30, 34, 35]. Although the exact effect of relaxation therapy in combination with other techniques is still unclear, our findings suggest that relaxation therapy might play an important role within multi-component treatment interventions. However, it is worthy of note that in one of the reviewed trials [23], the taught relaxation therapy had no statistical benefit over the control group, in which participants were simply told to relax. This could indicate that although relaxation is effective per se, it is not the expert-taught techniques or the hypnotherapy protocol which is beneficial, but simply taking the time to consciously relax the body on a regular basis. These observations could have practical clinical relevance, in consideration of the lack of trained therapists as a limiting factor for the implementation of behavioural treatment interventions on a larger scale [36].

As TS often presents with co-morbid conditions such as obsessive-compulsive disorder and attention-deficit and hyperactivity disorder [37, 38], it would be important to assess the effectiveness of relaxation therapy as a possible treatment intervention for TS-related conditions. The presence of co-morbid behavioural symptoms has been shown to have far-reaching consequences in terms of patients’ health-related quality of life [39, 40]. The results of three recent randomized controlled trials have suggested that cognitive behavioural therapy is more effective than relaxation therapy for the treatment of both obsessive-compulsive disorder [41, 42] and attention-deficit and hyperactivity disorder [43].

The present systematic literature review has limitations. The reviewed controlled trials had relatively small sample sizes, as well as heterogeneous study protocols, resulting in increased risk of bias and limited generalizability of the findings. Moreover, the majority of the studies included in the review were older, the oldest having been published in 1978, meaning there may be some discrepancy in validity between studies. The diagnostic criteria for TS have changed over time, raising the possibility that the results of some of the older studies might not be transferable or reproducible in patients who were diagnosed more recently [44]. Finally, the reviewed narrative case studies have a higher risk of both publication bias and sampling bias, because of the selective attention to patients who have proven unresponsive to other treatment interventions.

Further research is needed to establish whether relaxation therapy is an effective treatment intervention for tics in the general population of patients with TS. Larger randomized controlled trial comparing relaxation therapy to a control of minimal treatment would be useful to understand whether relaxation therapy is an effective intervention either in isolation or in conjunction with other types of behavioural therapy currently in use. Other areas for future research include the evaluation of the efficacy of relaxation therapy in comparison to other behavioural treatments and the identification of patient groups who are most likely to benefit from relaxation therapy, based on age, tic severity, presence of co-morbid conditions, and other clinical parameters.

In summary, the results of this systematic literature review showed preliminary evidence for the safety and efficacy of relaxation therapy as a treatment for tics in patients with TS. As the available literature is limited, further research is needed to confirm our preliminary findings and reach more accurate conclusions. Specifically, it is still unclear how relaxation therapy compares to other behavioural treatment interventions for tic control, as well as its exact role within multi-component behavioural interventions for tic control such as the Comprehensive Behavioral Intervention for Tics. The ongoing search for more effective treatment strategies is a key component of clinical research aimed at improving health-related quality of life in patients with tic disorders.
